# Reusable Flexible Concentric Electrodes Coated With a Conductive Graphene Ink for Electrotactile Stimulation

**DOI:** 10.3389/fbioe.2018.00179

**Published:** 2018-12-03

**Authors:** Benjamin Stephens-Fripp, Vitor Sencadas, Rahim Mutlu, Gursel Alici

**Affiliations:** ^1^School of Mechanical, Materials, Mechatronic and Biomedical Engineering, University of Wollongong, Wollongong, NSW, Australia; ^2^ARC Centre of Excellence for Electromaterials Science, University of Wollongong, Wollongong, NSW, Australia

**Keywords:** dry electrode, electrocutaneous stimulation, 3D printed electrode, graphene, conductive ink, sensory feedback

## Abstract

Electrotactile stimulation is a highly promising technique for providing sensory feedback information for prosthetics. To this aim, disposable electrodes which are predominantly used result in a high environmental and financial cost when used over a long period of time. In addition, disposable electrodes are limited in their size and configurations. This paper presents an alternative approach based on a 3D printed reusable flexible concentric electrode coated with a conductive graphene ink. Here, we have characterized the electrode and demonstrated its effective performance in electrotactile stimulation and sensory feedback for robotic prosthetic hands.

## Introduction

Despite the recent advances in the design and control of upper limb prosthetic devices on the marketplace (Atzori and Müller, 2015; Meek, 2017) and extensive research conducted on sensory feedback techniques (Stephens-Fripp et al., 2018a), the prosthetic devices are yet to incorporate sensory feedback into their functions. This lack of sensory feedback contributes to the high rejection (Biddiss and Chau, 2007a,b) and low usage (Davidson, 2002) of prosthetic devices and decreases the user's sense of embodiment (Ehrsson et al., 2008; Schmalzl et al., 2014; D'Alonzo et al., 2015). Prosthetic users have also shown a desire to incorporate sensory feedback to reduce their reliance on visual information (Atkins et al., 1996), as sight alone does not provide sufficient information for effective control (Johansson and Flanagan, 2009) and increases the congitive load on the amputee.

Electrotactile stimulation is a potential technique for providing sensory feedback information (Stephens-Fripp et al., 2018a). It is small and lightweight, contains no moving parts and requires a smaller amount of power compared to mechanotactile (Stephens-Fripp et al., 2018b) and vibrotactile feedback (Stephens-Fripp et al., 2018c). Further, it has a potential for a higher available bandwidth to communicate information (Szeto and Saunders, 1982) due to the multiple parameters of pulse width, frequency, amplitude, and location of stimulation being available for reliable manipulation.

Currently, disposable electrodes are the main type of electrodes used in sensory feedback research. Although some flexible electrode arrays have been developed (Isaković et al., 2016; Strbac et al., 2016), no reusable combined electrodes have been found within literature at the time of writing to the best of the author's knowledge. Although commercial dry electrodes are used in Transcutaneous Electrical Nerve Stimulation (TENS) stimulation for physiotherapy and pain relief purposes, they are typically larger in size and are not typically used within literature for providing sensory feedback, particularly when with multiple channels. In this work, we present an alternative to disposable electrodes, by developing a flexible, conductive, and reusable concentric electrode that can be used for electrotactile stimulation for sensory feedback. Although some research uses separate anode and cathode electrodes (Stephens-Fripp et al., 2018a), concentric electrodes are the preferred design for sensory feedback as they result in better localization of the induced sensation (Szeto and Saunders, 1982; Szeto and Riso, 1990; Isaković et al., 2016; Strbac et al., 2016), and minimize the interference with electromyography (EMG) (Jiang et al., 2014), which is often used in the control of prosthetic devices.

Flexible reusable electrodes have been previously developed (Rogers et al., 2010; Lepola et al., 2014; Khan et al., 2016), for applications such as electroencephalogram (EEG), electrocardiogram (ECG), and electromyography (EMG). These electrodes are typically smaller in size to offer higher resolution in signal recognition. However, using electrical stimulation for the purpose sensory feedback requires larger electrodes to produce a comfortable sensation (Kuhn et al., 2010; Gomez-Tames et al., 2012; Li et al., 2015). In addition, the high impedance value for electrical stimulation (Lepola et al., 2014), or the conductive material based in sputtered metals reduces its stretchability (Khan et al., 2016), and therefore loses its flexibility when a larger surface area is required.

Due to the wastage of materials, cost, skin irritation, and signal degradation over time resulting from the use of disposable electrodes, several studies have researched viable reusable replacement electrodes. Polymers mixed with either silver microparticles or carbon additives have been considered for their application in ECG and EEG recordings (Chen et al., 2014; Stauffer et al., 2018). Rubber and fabric-based materials have also been examined for creating flexible reusable electrodes in EMG signal detection (Pylatiuk et al., 2009). Krachunov and Casson used 3D printing to create rigid dry EEG electrodes and painted them with a silver coating to increase their conductivity. For electrical stimulation on the forearm, however, flexibility is important to conform to the surface of the arm and skin.

In this paper, we present the process of coating a 3D printed flexible substrate with a thin layer of conductive graphene ink to create a low-cost reusable flexible electrode that can be used in the application of electrotactile stimulation without the need for additional adhesive.

## Electrode development

**Figure 1:**
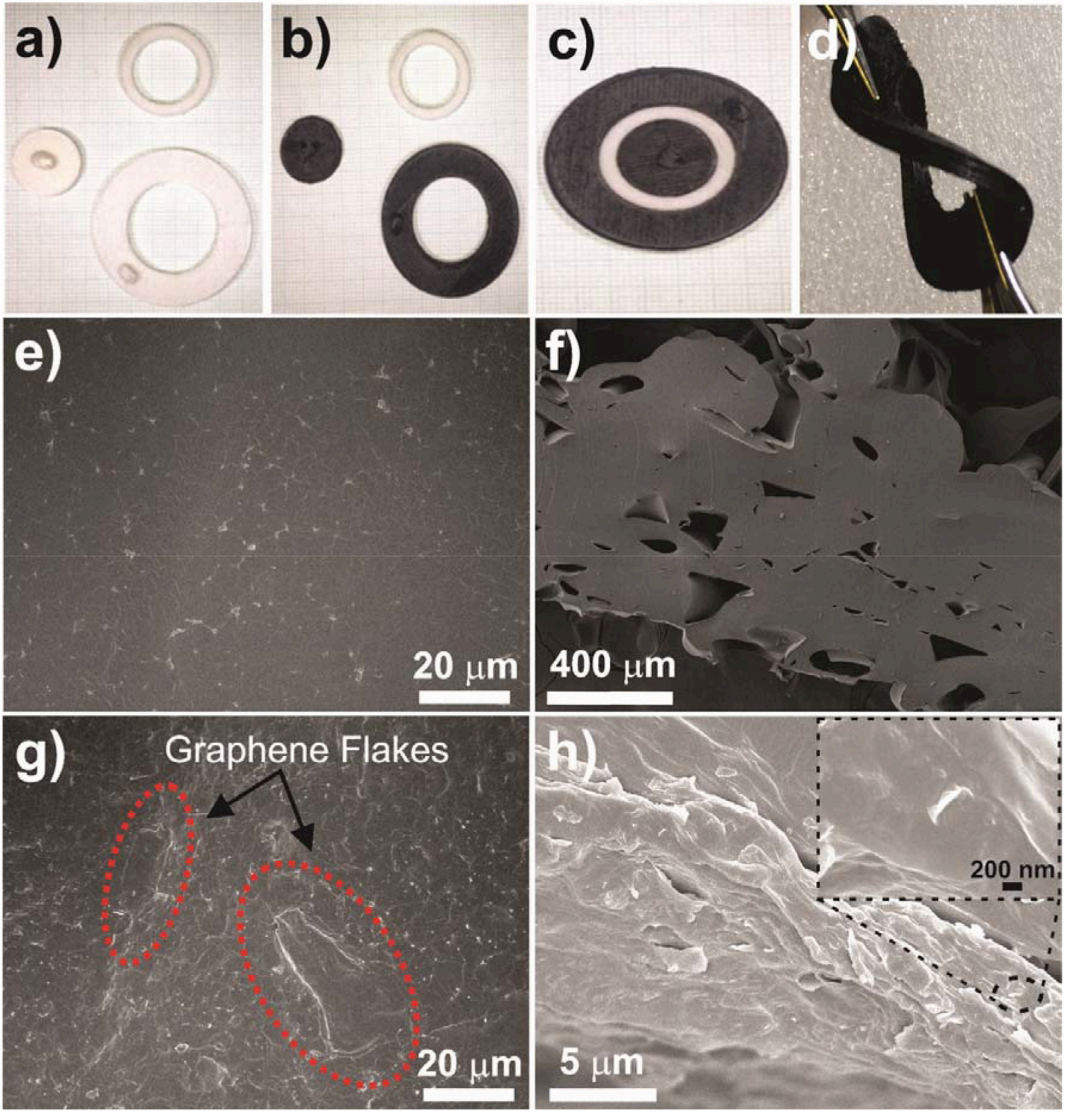
Electrode (on 1 mm grid paper): **(a)** 3D printed uncoated electrode components, **(b)** coated electrode components, **(c)** assembled electrode, **(d)** demonstration of coated electrode's flexibility, **(e)** surface and **(f)** cross-section morphology of the pristine 3D printed part, **(g)** surface of the spray coated layer, and **(h)** detail of the interface between the spray coated layer and the 3D printed component. Inset shows a graphene flake on the spray coated layer.

The basic electrode structure was 3D printed (Flashforge Inventor) using Ninjaflex material in three sections; inner electrode, separator, and outer electrode; as shown in Figure 1a. The print was performed using a layer height of 0.18 mm, a fill density of 35%, and an overlap of 30%. The inner electrode has a diameter of 15 mm to match the size of the disposable electrode and produce an equivalent current density. The separator has a width of 5 mm, and the outer electrode has an inner diameter of 20 mm and an outer diameter of 35 mm. This outer electrode size was chosen from initial testing of the electrotactile stimulation to produce a comfortable sensation. Flexibility of the 3D printed electrode is demonstrated by clamping across the electrode and twisting it as, shown in Figure 1d, demonstrating that the electrode can undergo high deformations with no permanent damage to either the structure or the conductivity of it. Due to this flexibility of the base material the components compress as they are pushed together and stay connected without the need for any adhesives. This allows for easy disassembly and reassembly for cleaning and sterilization. All three sections have a 3 mm thickness to provide an effective compression fit when pushed together, as shown in Figure 1c. The inner and outer electrodes have a knob on top (4 × 2 × 3 mm high, Figures 1a–d) to allow easy attachments to the electrical stimulator and other measurement and testing devices.

Graphene flakes (5 μm particle size, surface area 120–150 m^2^/g, Sigma-Aldrich) were dispersed by probe sonication (Bandelin UW 3200) in toluene (anhydrous, 99.8%, Sigma-Aldrich) for 60 min. Afterwards, styrene-ethylene-butadiene-styrene (SEBS, Calprene CH6120, Dynasol Gestion, S.A.) was added to the solution. The dissolution process was performed at room temperature using a magnetic stirrer (MST, Velp Scientifica) until complete polymer dissolution. The solution was then transferred to a spray gun chamber to spray it against the surface of the 3D printed parts, followed by allowing the solvent evaporation at room temperature.

The ratio between the polymer and solvent used was 5 wt% polymer in 95% solvent, and the amount of conductive filler present in the dry polymer layer was 4 wt%. Sample morphology was assessed by scanning electron microscopy (SEM, JSM-7500FA, JEOL). The samples were previously coated with a platinum thin layer (~10 nm) by sputtering. Raman spectra were collected with a Horiba Jobin Yvon LabRAM HR 800 Raman spectrometer using excitation of He-Ne 632.8 nm with a power 13.5 mW.

The sheet resistance was measured by a four-point probe system (Jandel RN3) using a square array probe with 0.635 mm spacing. Ten readings were taken, measuring both the forward and reverse current from five different locations, and the average sheet resistance was calculated across these 10 samples.

Impedance measurements were taken using an MFIA Impedance Analyzer (Zurich Instruments) from 1 kHz to 1 MHz.

## Electrode characterization

Figures 1e,f presents the microscopic morphology of the 3D printed parts. While the surface presents some roughness characteristic of the 3D printed lines, no voids, or microscopic pores where visible (Figure 1e), on the other hand, the cross-section of the 3D printed parts showed some voids between the different printed lines (Figure 1f) that such porosities improve flexibility of the structure of the electrode (i.e., 3D printed part).

When the conductive solution is sprayed on the surface of the pristine 3D printed parts, a layer of SEBS with graphene flakes was covering all the parts uniformly (Figure 1g). Moreover, it was noticed the absence of graphene flakes clusters, suggesting that the dispersion method was efficient in destroying the conductive filler aggregates and creating a homogeneous solution.

The adhesion between the SEBS and the 3D parts is quite strong and it was difficult to peel-off the conductive layer from the supporting part (Figure 1h).

Toluene is a weak solvent for Ninjaflex, and when the sprayed droplets of conductive solution contacts the surface of the polymer substrate, the toluene will be partially up taken, promoting dissolution of the polymer chains at the surface of the 3D printed part, and allowing the SEBS to blend with the Ninjaflex chains. Sencadas et al. (2017) reported that the toluene promotes chemical affinity between the styrene-butadiene-styrene (SBS) and the thermoplastic urethane (TPU) substrate. In their work, it was shown that the solvent can promote partial dissolution of the polymer chains at the surface of the TPU 3D printed part, where the SBS blended, creating a compliant strain sensor.

To enable easy application to the human arm, an off the shelf conductive TENS adhesive (TAC GEL) was applied to the bottom surface of both the graphene coated sections of the electrode. This enables the electrodes to stick to the arm without the need for tape. However, demonstration testing was conducted both with and without the conductive gel to compare the electrode performance.

### Raman analysis

**Figure 2:**
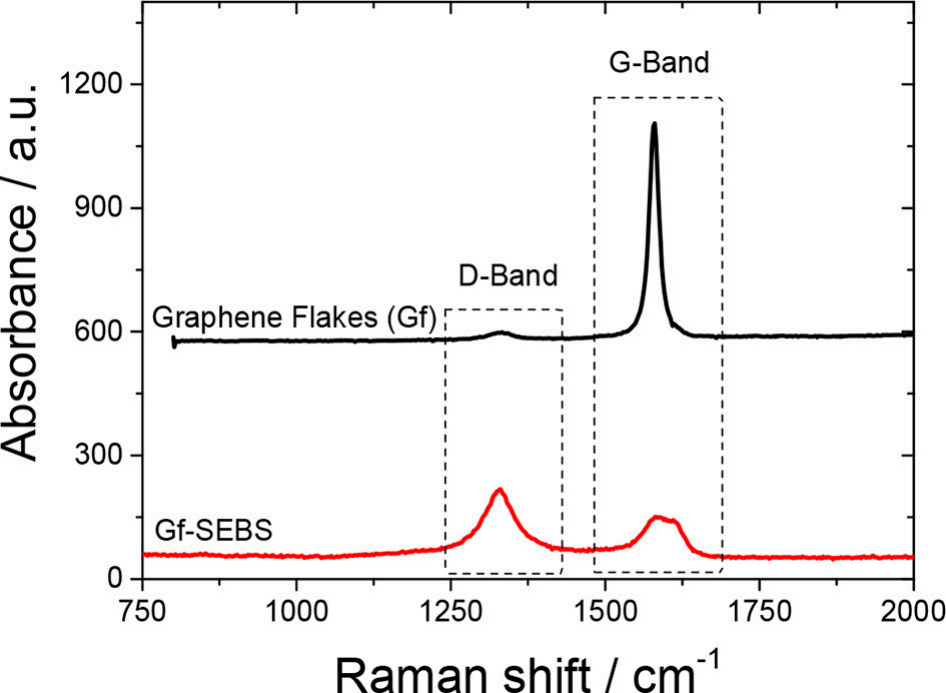
Raman spectra collected for the graphene flakes and Gf-SEBS spray coated layer.

The Raman spectra of the graphene flakes used and the Gf-SEBS layer is presented in Figure 2. Gf present the characteristic absorption band of the G-band at 1,580 cm^−1^, assigned to the graphite-like tangential mode (Papageorgiou et al., 2015). Moreover, for the Gf-SEBS sprayed sample, an increase in the absorption band at 1,360 cm^−1^, assigned to the D-band of the graphene flakes, which corresponds to the impurities or defects of the conductive filler (Papageorgiou et al., 2015; Yan et al., 2018a).

The disorder in graphene layers is usually qualitatively analyzed by the $\frac{I_{D}}{I_{G}}$ intensity ratio between the disorder D-band and the G-band. Figure 2 shows that the incorporation of the graphene flakes into the SEBS solution and sprayed on the surface of the Ninjaflex 3D printed components led to an increase in the $\frac{I_{D}}{I_{G}}$ ratio, from 0.03 up to 1.65, suggesting that the ultrasonication process promotes disordering in the bond strengths (Ferrari and Robertson, 2000; Ado et al., 2010), also observed by the broadening of the G-band absorption (Figure 2). The creation of more disordered graphene flakes could promote chemical conjugation between the Gf filler, SEBS, and Ninjaflex polymer chains. Furthermore, the broadening of the D-band for the Gf-SEBS layer sample suggests the formation of covalent bonds between the SEBS and the filler (Ferrari and Robertson, 2000; Chen et al., 2005; Ado et al., 2010; Yan et al., 2018a,b).

### Sheet resistance

The average sheet resistance of the graphene coated electrode across the 10 readings taken was determined to be 903.5 ± 262.15 Ω/□.

Since the sheet resistance is a characteristic used to compare the conductivity of thin materials, it would be invalid to measure the conductive material in the disposable electrode due to its large thickness. Therefore, conductivity comparisons between the disposable electrode and graphene based electrode will be instead made from the impedance measurements as shown in section Impedance Measurements.

Even though the sheet resistance is unable to be used to compare performance against the disposable electrode, the authors felt that the sheet resistance is a useful measurement to include to help readers understand the properties of the graphene coating. In addition, it will provide a benchmark for comparison against future research on the development of flexible resusable electrodes.

### Scratch test

**Figure 3:**
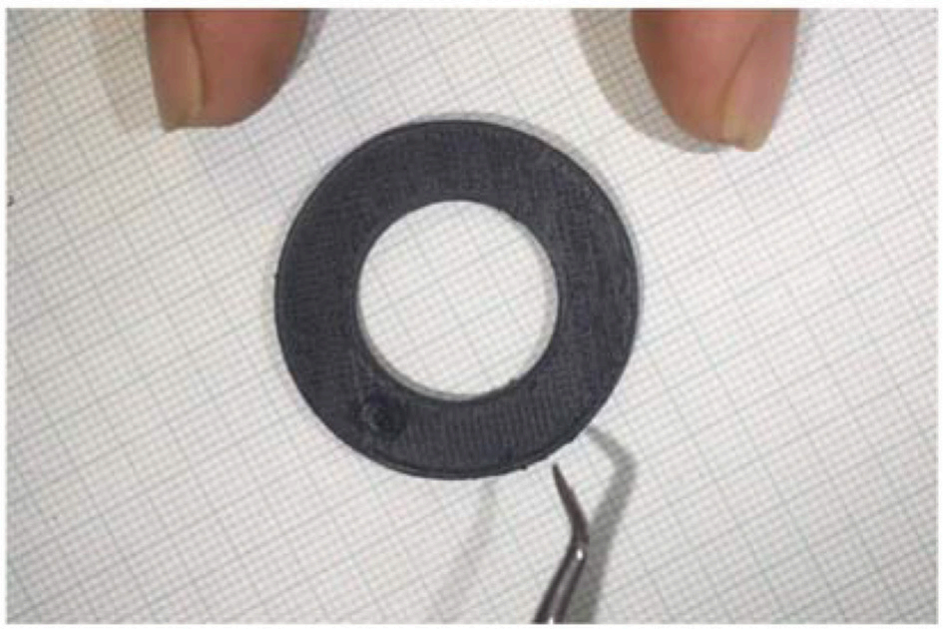
Scratch test performed with hook tool.

To ensure robust adhesion of the graphene coating, a scratch test was performed. This was conducted by scraping the electrode with a hook tool, shown in Figure 3, followed by a pair of tweezers. After both scraping sessions, no marks, or damage was visible on the electrode and no change in impedance was recorded. A video of this process can be found in the Supplementary Material.

### Environmental and financial cost

In addition to providing more versatility in custom electrode design, this electrode design has potential to have a financial saving and significant reduction of the environmental impact of regularly using disposable electrodes.

In this analysis, we base our calculations off a batch of ten concentric electrodes being produced at once, which in addition to resting and drying time, requires 2 h of ink preparation and roughly 10 min to spray. This equates to ~13 min of preparation time per electrode, which would reduce when making a larger batch as there would be a minimal increase in ink preparation time. Table 1 outlines the costs of the materials required for both printing the base material and the ink coating. This does not consider the cost of equipment required for ink preparation/spraying or 3D printing. The largest cost of the electrode is from the Ninjaflex filament, which could be reduced in size, particularly in developing thin flexible electrodes to be embedded in a fabric.

**Table 1:** Material costs of concentric 3D printed electrode for a batch of 10 concentric electrodes.

**Material and Price**	**Amount required**	**Price per batch ($)**
SEBS—$0.5/1 kg	0.5 g	0.0025
Toluene—$73.5/L	3 mL	0.2205
Graphene—$50/5 g	20 mg	0.2
Ninja Flex $93/750 g	3.3 g per concentric electrode	4.11
Total material cost per batch		$4.53
Total material cost per electrode		$0.45

Based off the durability of the electrode demonstrated in the scratch test, and the known flexible properties of the Ninjaflex materials, a 1-year life-time is estimated for the custom printed flexible concentric electrode. Further analysis and testing are required to determine any reduction in performance or durability over longer periods of time and repeated use. Within this period of time, using one pair of disposable electrodes per day would result in a total use of 730 electrodes. At an approximate costing of $1.30 per a disposable electrode (Core Electonics, 2018), using the concentric electrode proposed in this study would result in a significant saving both financially and environmentally as a result of the reduction in waste produced.

### Impedance measurements

**Figure 4:**
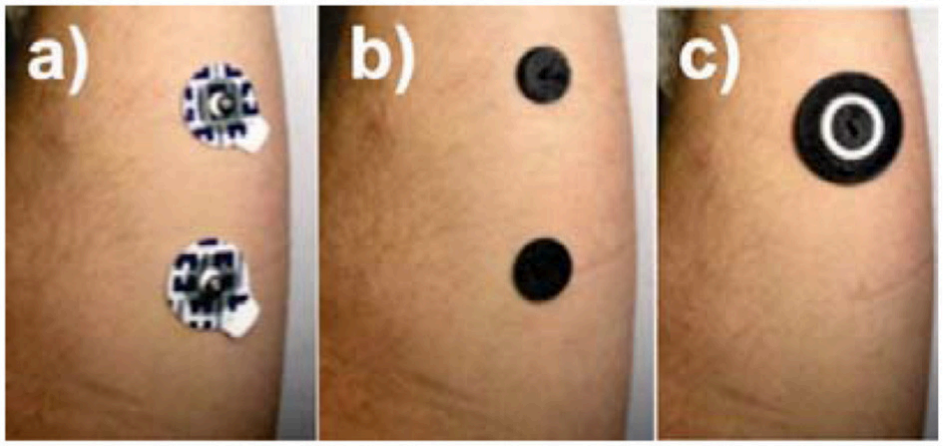
Positioning of electrodes for impedance test: **(a)** dual disposable electrodes, **(b)** dual 15 mm graphene coated electrodes (shown here with adhesive), and **(c)** concentric graphene coated electrodes (shown here with adhesive).

Due to the different locations that result from placing a concentric electrode (Figure 4c) compared to disposable electrode pairs (Figure 4a), it would be invalid to compare impedances between the two. Therefore, an additional test was conducted using dual graphene electrode pairs (Figure 4b). This was to enable a comparison based on the material properties and the electrode geometrical configuration. Five different electrode combinations were, therefore, tested for comparison: 15 mm disposable electrode pairs (Figure 4a); dual 15 mm graphene covered electrode pairs (Figure 4b), tested dry and with conductive adhesive; graphene coated concentric electrode (Figure 4c), tested dry, and with conductive adhesive.

**Figure 5:**
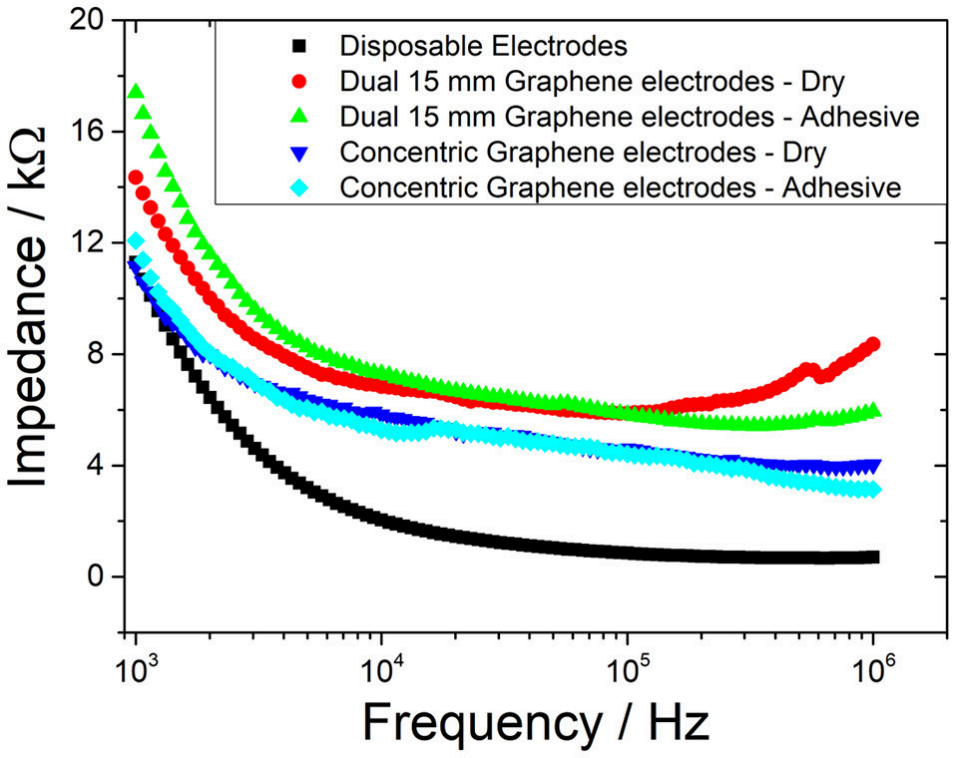
Impedance measurement from 1 kHz to 1 MHz.

Typical pulse width range used in electroctactile stimulation for prosthetic sensory feedback ranges from as low as 50 μs up a value of 500 μs (Xu et al., 2016). Therefore, the frequency band of interest is 1–10 kHz. As shown in Figure 5, although the disposable electrode's impedance values were slightly higher, the graphene-coated electrodes are comparable within this frequency range. In addition, the concentric configuration (Figure 4c) also slightly reduced the impedance of the electrode; however, this would be largely due to the fact that the current flows through a smaller distance within the body.

Testing in this study was conducted at a pulse width of 100 μs which corresponds to a frequency of 5 kHz. At this frequency, the corresponding impedance is ~3.2 kΩ for the disposable electrodes, ~6.2 kΩ for the concentric graphene electrodes, and ~8 kΩ for the dual graphene electrodes.

## Application demonstration

The focus of this electrode design is for the use in electrotactile stimulation, which is demonstrated in section Electrical Stimulation. However, to show the potential use of these electrodes in a wide range of applications, section EMG and ECG Recording demonstrates the electrode's use in EMG and ECG signal recording. Written informed consent was obtained from the individual participating in this study and ethical approval was obtained from the University of Wollongong Human Research Ethics Committee.

### Electrical stimulation

The electrodes were tested in two stages: determining the range of comfortable stimulation current, followed by recording the current flowing through the electrode. For both experiments, stimulation was provided through a BioPac constant current linear isolated stimulator (STMISOLA) controlled through a Biopac MP36 data acquisition system. Stimulation was provided through a biphasic square wave with a pulse width of 100 μs, frequency of 10 Hz and an inter-pulse delay of 100 μs.

#### Range of sensation

As used in the impedance measurement, five electrode combinations were used and in the same locations shown in Figure 4. The stimulation current was slowly increased from 0 mA by increments of 0.1mA until the subject indicated a perception of the electrotactile stimulation. This value was recorded as the minimum perception threshold. The current amplitude was then slowly increased by increments of 0.1 mA until the stimulation current became uncomfortable or resulted in muscle twitching. The stimulation was then stopped, and this current was set as the maximum threshold. After a 5-min break, this process was repeated with the next electrode type. These tests were repeated three times with the electrodes in a different order to ensure no variance due to adaptation to the electrical stimulation had occurred. The results are shown in Table 2, and there was no variability detected between the three different tests. Using the impedance measurements at 500 kHz, duty cycle of 2% (100 Hz, PW 100 μs), the RMS Power for the minimum perception and maximum comfortable threshold is also displayed in Table 2.

**Table 2:** Range of comfortable perception of electrotactile stimulation of the different electrodes—Frequency 100 Hz, Pulse Width 100 μs, Inter-Pulse Delay 100 μs.

**Electrode type**	**Impedence (kΩ)**	**Minimum perception threshold (mA)**	**Average power @ 2% duty cycle (mW)**	**Maximum comfortable threshold (mA)**	**Average power @ 2% duty cycle (mW)**
Dual disposable electrodes	3.2	2.5	0.40	4.5	1.30
Dry dual 15 mm graphene coated electrodes	8	2.5	1.00	4.5	3.24
Dual 15 mm graphene coated electrodes with conductive adhesive	8.6	2.5	1.08	4.5	3.48
Dry concentric graphene coated electrode	6.3	2.7	0.92	5.4	3.67
Concentric graphene coated electrode with Conductive Adhesive	6	2.7	0.87	5.4	3.50

#### Stimulation current waves

Although the stimulator produces square waves, due to the capacitance of the skin and the electrode, the transmitted waveforms have an associated rise time and do not form perfect square waves. To view these current waveforms flowing through skin, the transmitted current was recorded using a National Instruments Current Input Module (NI-9203). A constant current biphasic square wave with a peak current of 4 mA was used for the electrotactile stimulation to ensure that it was within the comfortable and recognizable range as shown in Table 2. The pulse width, frequency, and inter-pulse delay were left at 100 μs, 100 Hz, and 100 μs, respectively.

**Figure 6:**
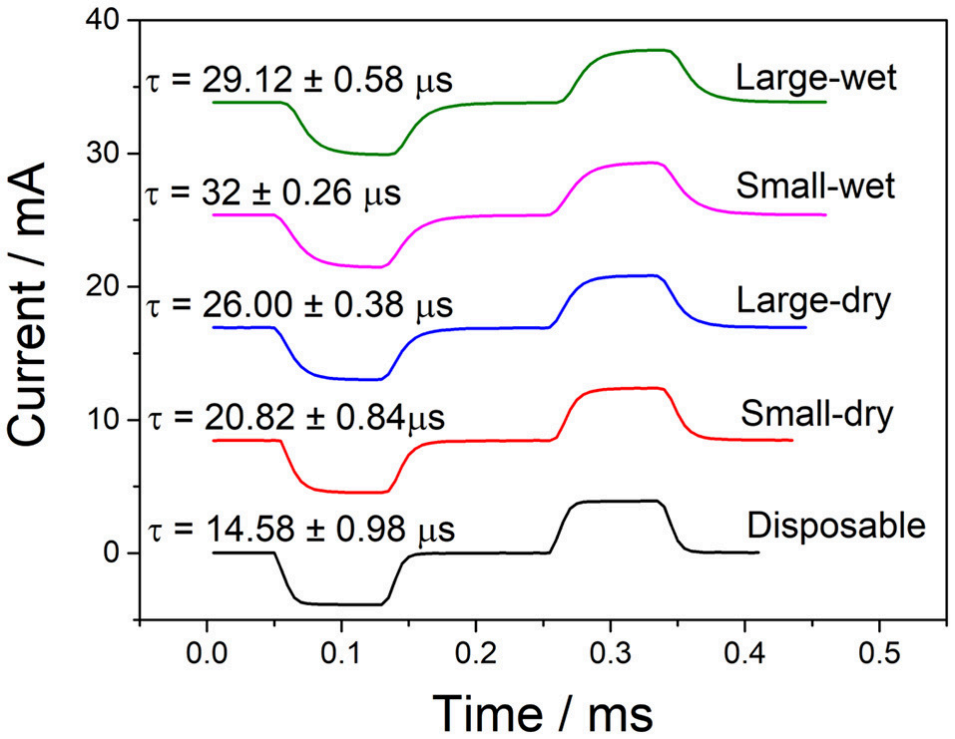
Measuring current from TENS stimulation through various electrodes (amplitude−4 mA, frequency−100 Hz, pulse width−100 μs, InterPulse delay−100 μs).

A single pulse for each electrode pair is shown in Figure 6, with their associated rise times averaged from five sequential pulses. Although the disposable electrode pair has a slightly lower time, all electrodes produce comparable wave forms with comparable rise times. It is also worth noting that since the current input module had a maximum sampling rate of 200 kHz, it was only able to take a current reading every 5 μs.

### EMG and ECG recording

The EMG and ECG data was obtained through the use of a BioPac MP36 data acquisition system. Both recordings were performed with 15 mm disposable electrodes, and then repeated with the graphene coated 15 mm electrodes for comparison. This process was repeated after 9 weeks to examine the longer term stability of the electrodes.

**Figure 7:**
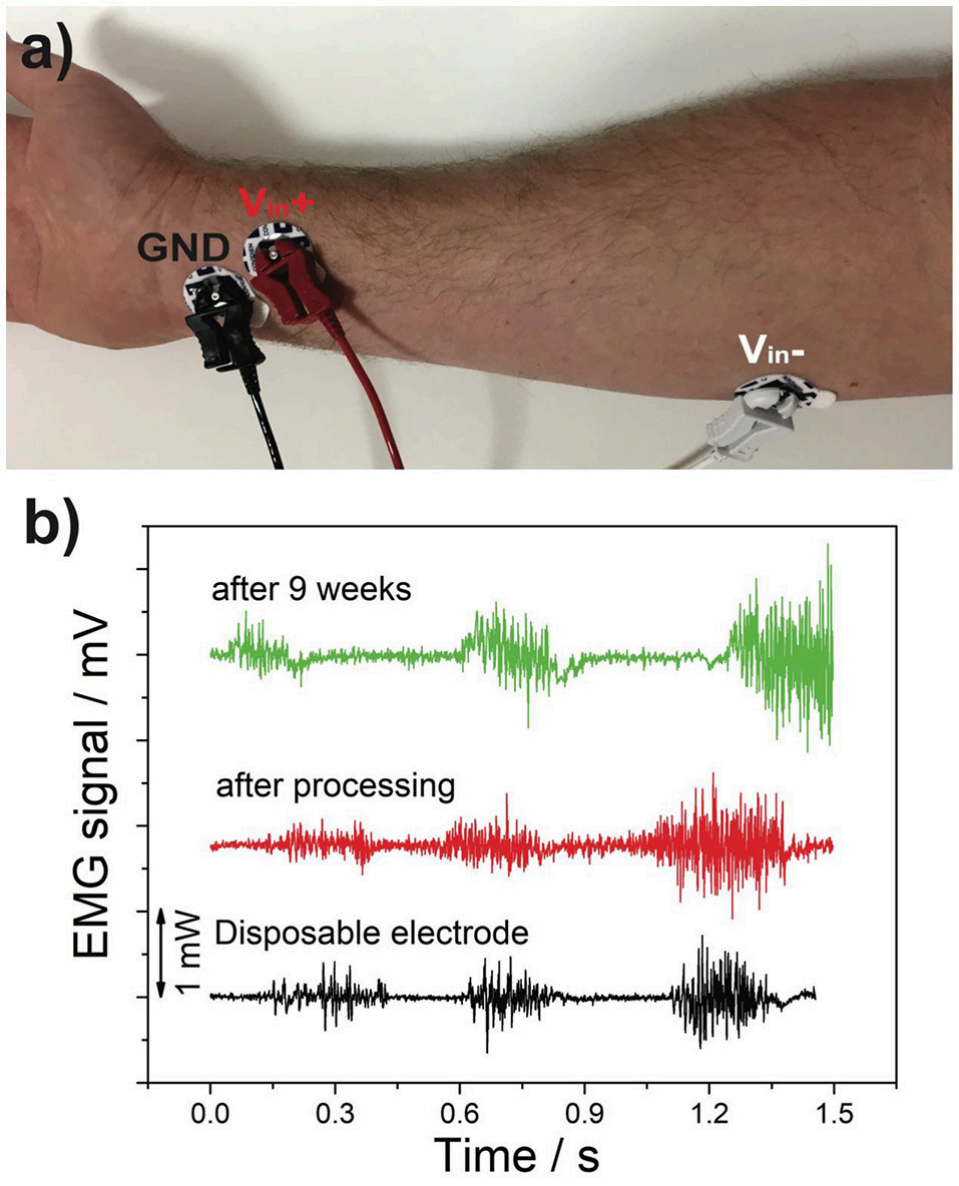
EMG signal recording. **(a)** Electrode Placement; **(b)** EMG Signal.

The EMG data, shown in Figure 7, was performed with the dominant arm being clenched at low, medium, and high intensity levels separated by a brief rest period. The graphene electrodes appear to have a higher level of noise in the rest period than that of the disposable electrodes. However, as can be seen in Figure 7, the electrode attachments are heavy which may have deteriorated the surface contact as there was no adhesive gel on the electrode which may have contributed toward the noise. The recording that was repeated after 9 weeks also showed no significant difference.

**Figure 8:**
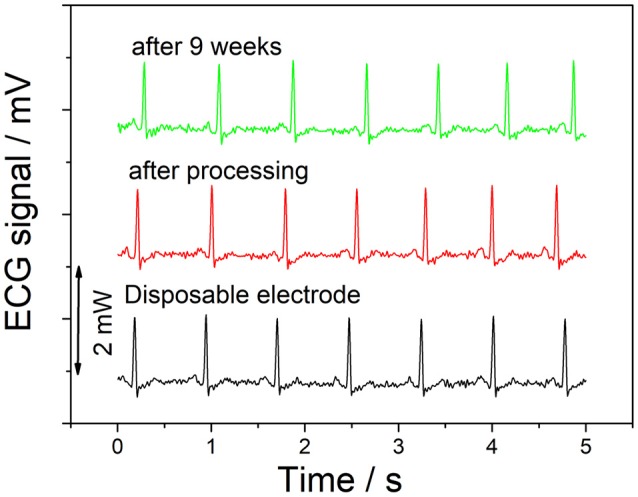
ECG signal recording.

The ECG signal was taken during a resting heart rate, with one electrode at each ankle, and one on the right forearm. As can be seen as Figure 8, there is no significant visible difference between the signals recorded using disposable electrodes and the graphene electrodes. In addition, the recording taken after 9 weeks also shows no degradation of the recorded signal.

## Discussion and conclusion

In this work, we have presented a proof of concept for a method to develop reusable flexible electrodes for electrotactile stimulation in providing sensory feedback to amputees using prosthetic devices. These electrodes allow for a cheaper and more environmentally friendly option for long-term use.

In our testing, these electrodes demonstrated a higher, but comparable impedance to that of disposable electrodes which have been previously used in literature. The higher impedance resulted in a higher voltage required to maintain the desired current. Although this would also result in an increase in the power consumption, with an effective duty cycle of 2% (for the 100 μs pulse width used), its impact would be minimal, as shown in Table 2.

The small layer of graphene was shown to sufficiently bond to the underlying thermoplastic, with its integrity remaining intact following the mechanical scratch test. This robustness is important for longer term use in prosthetic applications.

Although the addition of conductive adhesive to the flexible electrodes made it easier to stay attached for testing purposes, there was no noticeable difference in performance between the graphene electrodes used dry or with the conductive adhesive. This allows for a future opportunity of the electrodes being built into fabrics that can be wrapped around the arms. Removing the adhesive that is often used in disposable and reusable electrodes, could reduce the level of irritation on the skin, and reduction in performance over time (Chi et al., 2010; Yao and Zhu, 2016). In addition, recent developments in electrotactile stimulators can tolerate differences in impedance that may result from a small movement of electrodes (Akhtar et al., 2018). Unfortunately, since the disposable electrode's gel has a larger diameter than the metal electrode insert, comparing its performance without the adhesive would not produce a fair comparison between the two. Furthermore, the conductive gel on the disposable electrode also conforms to the surface of the skin, creating a better contact between them. This may help decrease impedance and increase performance. Therefore, investigating the use of a conforming conductive layer between the 3D printed electrode and the surface of the skin may result in an increase in the electrode's performance and electrical efficiency. Alternatively, previous studies have indicated that the impedance of dry electrodes can be reduced by applying additional pressure as it helps increase the surface contact area with the skin (Gruetzmann et al., 2007; Myers et al., 2015). Analysis is therefore required to find the optimum pressure and its impact of impedance in these concentric electrodes.

Further testing is required to determine the optimum geometry and sizing of these electrodes. Since their shape is manufactured through additive manufacturing, it allows custom printing of the electrodes to match the curvature of the arm through a 3D body scanner. The current electrode was printed with a 3 mm thickness, to enable a compression fit between the different electrode layers. However, if they were instead built into a fabric or other surface layer, this compression fit would no longer be required. This would enable the electrodes to be printed significantly thinner, which would further increase the flexibility, and electrodes allow for better conformance to surface of the human arm.

Our testing of stimulation using the concentric electrodes showed a higher range of a wave current that was tolerated by the user. We postulate that this is primarily due to the lower current density on the skin arising from the larger surface area of the outer electrode. However, further testing and analysis are required to examine this effect comprehensively and determine the optimum geometry. In addition, psychometric analysis is required not only to better compare the perception ranges, but also the just noticeable differences of the stimulation properties to determine the identifiable levels of stimulation obtainable in each electrode.

Although the focus of this paper has been on the use of these electrodes for electrotactile stimulation for prosthetic sensory feedback, they could potentially be also used in signal detection or retrieval, such as in EMG recognition used in prosthetic control. Even with higher impedance values, dry electrodes can result in a lower signal to noise ratio (SNR) in EMG recognition than commercially available gelled electrodes (Myers et al., 2015). However, further research is required to measure the electrode's performance in signal retrieval and optimizing the electrode's size, shape, and geometry for this application.

## Ethics statement

This study was carried out in accordance with the recommendations of Australian National Statement on Ethical Conduct in Human Research. The protocol was approved by the University of Wollongong Human Research Committee. All subjects gave written informed consent in accordance with the Declaration of Helsinki.

## Author contributions

BS-F designed the electrode shape and printed the base electrode. VS developed and applied the graphene coating to the electrodes, wrote the graphene preparation and application, as well as the Raman Analysis and Electron Microscrope sections of the manuscript. BS-F conducted all testing procedures and wrote the draft of themanuscript. BS-F, VS, RM, and GA contributed to the conception and design of the study. All authors contributed to manuscript revision, read, and approved the submitted version. GA supervised the project.

### Conflict of interest statement

The authors declare that the research was conducted in the absence of any commercial or financial relationships that could be construed as a potential conflict of interest.

## Supplementary material

Supplementary Video 1 Electrode scratch test video.
